# Clinical significance of COX-2, GLUT-1 and VEGF expressions in endometrial cancer tissues

**DOI:** 10.12669/pjms.312.6604

**Published:** 2015

**Authors:** Xiaoping Ma, Yuzuo Hui, Li Lin, Yu Wu, Xian Zhang, Peishu Liu

**Affiliations:** 1 Xiaoping Ma, Department of Gynecology and Obstetrics, Qilu Hospital Affiliated to Shandong University, Jinan 250012, Shandong Province, P.R. China. Liaocheng People’s Hospital and Clinical Teaching Hospital, Taishan Medical College, Liaocheng 252000, Shandong Province, P.R. China; 2 Yuzuo Hui, Department of Neurosurgery, Liaocheng People’s Hospital and Clinical Teaching Hospital, Taishan Medical College, Liaocheng 252000, Shandong Province, P.R. China; 3 Li Lin, Department of Gynecology and Obstetrics, Qilu Hospital Affiliated to Shandong University, Jinan 250012, Shandong Province, P.R. China. Liaocheng People’s Hospital and Clinical Teaching Hospital, Taishan Medical College, Liaocheng 252000, Shandong Province, P.R. China; 4 Yu Wu, Department of Gynecology and Obstetrics, Liaocheng People’s Hospital and Clinical Teaching Hospital, Taishan Medical College, Liaocheng 252000, Shandong Province, P.R. China; 5 Xian Zhang, Department of Gynecology and Obstetrics, Liaocheng People’s Hospital and Clinical Teaching Hospital, Taishan Medical College, Liaocheng 252000, Shandong Province, P.R. China; 6 Peishu Liu, Department of Gynecology and Obstetrics, Qilu Hospital Affiliated to Shandong University, Jinan 250012, Shandong Province, P.R. China. Liaocheng People’s Hospital and Clinical Teaching Hospital, Taishan Medical College, Liaocheng 252000, Shandong Province, P.R. China

**Keywords:** Endometrial cancer, COX-2, GLUT-1, VEGF

## Abstract

**Objective:**

To analyze the clinical significance of COX-2, GLUT-1 and VEGF expressions in endometrial cancer tissues.

**Methods:**

One hundred and eight tissue samples from the patients with endometrial cancer enrolled in our hospital from August 2011 to July 2014 were selected, including 60 normal tissue samples (normal group), 60 neoplastic tissue samples (neoplastic group) and 60 cancer tissue samples (cancer group). All the samples were subjected to immunohistochemical assay to detect the expressions of COX-2, GLUT-1 and VEGF. The clinical data were also investigated for correlation analysis.

**Results:**

The positive rates of COX-2 in normal group, neoplastic group and cancer groups were 3.3%, 21.7% and 55.0% respectively. The positive rates of GLUT-1 in normal group, neoplastic group and cancer groups were 3.3%, 25.0% and 70.0% respectively. The positive rates of VEGF in normal group, neoplastic group and cancer groups were 1.7%, 23.3% and 63.3% respectively. With increasing stage of such cancer, decreasing degree of differentiation and lymphatic metastasis, the positive expression rates of COX-2, GLUT-1 and VEGF proteins were raised significantly (P<0.05). Spearman’s correlation analysis showed that the expressions of COX-2 and GLUT-1 (r=0.207, P<0.05), COX-2 and VEGF (r=0.243, P<0.05), as well as GLUT-1 and VEGF (r=0.758, P<0.05) were positively correlated.

**Conclusion:**

COX-2, GLUT-1 and VEGF were highly prominent in endometrial cancer, especially in the patients with low degree of differentiation, late stage and metastasis. They functioned synergistically in the onset and progression of this cancer.

## INTRODUCTION

Endometrial cancer, as one of the common malignant cancers of the female reproductive system, is threatening more women worldwide, even the young ones.^1^ The pathogenesis of endometrial cancer remains unclear hitherto, including a multi-step, multi-stage, multi-factor biological evolution process that involves a number of genetic variations.^2^,^3^ Recently, the carcinogenic mechanism of endometrial cancer has been explored by using molecular genetics, molecular biology in combination with immunological techniques.^4^,^5^ In the viewpoint of molecular biology, endometrial cancer may be attributed to uncontrollable cell proliferation-induced malignant transformation that results from abnormal activation of multiple oncogenes, over expression of encoded proteins, as well as deletion, mutation and inactivation of anti-oncogenes.^6^

It has previously been reported that epidermal growth factor receptor and vascular endothelial growth factor (VEGF) play important roles. Particularly, the regulation of VEGF gene expression is associated with many factors such as differentiation, hormones, cytokines and partial pressure of oxygen.^7^ As the most potent vascular endothelial cell division-promoting agent, VEGF is key for the onset, invasion and metastasis of tumors, which prevents tumor cells from immune response by facilitating tumor growth and by hindering maturation of host-specific antigen-presenting cells.^8^

Currently, glycometabolism has been widely related with the onset and progression of tumors. In particular, human glucose transporter-1 (GLUT-1), which belongs to the family of activated glucose transporter proteins, plays a crucial role in the glucose uptake of many types of tumors. GLUT-1 is highly expressed in endometrial cancer, which can be used to differentiate benign endometrium from atypically hyperplastic endometrium.^9^ Over expression of GLUT-1 enhances the metabolism of tumors and accelerates their proliferation, thus energizing the variation, differentiation and growth of tumor cells.^10^

Cyclooxygenase-2 (COX-2), also known as prostaglandin-endoperoxide synthase, has stable mRNA and protein levels in human body, mainly distributed in the endoplasmic reticulum to promote the synthesis of prostaglandin and to maintain normal functions of human body. However, it is usually highly expressed during the inflammation of most tissues and in tumors. Meanwhile, in endometrial cancer tissues, activation of oncogenes, mutation of anti-oncogenes and abnormal cell proliferation may be inter-connected in a complex paradigm.^11^,^12^

In this study, we analyzed the expressions of COX-2, GLUT-1 and VEGF in endometrial cancer, aiming to evaluate their clinical significance in the onset, progression, infiltration and metastasis of this cancer, and to clarify their correlations.

## METHODS

### Subjects

One hundred and eight tissue samples from the patients with endometrial cancer enrolled in our hospital from August 2011 to July 2014 were selected, including 60 normal tissue samples (normal group), 60 neoplastic tissue samples (neoplastic group) and 60 cancer tissue samples (cancer group).

### Inclusion criteria

With completed clinical data; aged 20-60 years old; squamous cell carcinoma; without receiving surgery, radiotherapy, chemotherapy or hormone therapy before being enrolled; with written consent form from all patients.

The normal group was aged 28-57 years old, with the average of (48.33 ± 3.11). The neoplastic group was aged 29-58 years old, with the average of (49.11 ± 2.87). The cancer group was aged 28-57 years old, with the average of (48.23 ± 4.11). FIGO staging: 34 case of Stage I, 20 cases of Stage II and 6 cases of Stage III; degree of differentiation: 21 cases of low differentiation, 19 cases of moderate differentiation and 20 cases of high differentiation. Lymphatic metastasis: 45 cases without and 15 cases with. The ages of the three groups were basically similar (P>0.05).

### Data survey

The gender, identification number, age, telephone, staging, degree of differentiation and lymphatic metastasis of the three groups were investigated. Meanwhile, the immunohistochemical staining results of COX-2, GLUT-1 and VEGF were recorded.

### Sample detection

All 4 μm-thick tissue samples were prepared by fixation in 10% formalin, routine dehydration, immersion in paraffin, embedding and continuous slicing sequentially. Then the sections were incubated with 3% H_2_O_2_ at room temperature for 10 minutes to remove endogenous peroxidase, and were subjected to antigen retrieval in a microwave at high temperature for 10 minutes. After deparaffinization by using dimethyl benzene, immunohistochemical staining was performed by adding different monoclonal antibodies (dilution, 1:1000), with PBS as the negative control to replace primary antibody. After being incubated at 4°C overnight, the sections were washed three times by PBS (5 min each), and immunohistochemical staining was conducted according to the manufacturer’s instructions before final sealing.

Rabbit anti-human COX-2 monoclonal antibody was purchased from DAKO, rabbit anti-human GLUT-1 immunohistochemical polyclonal antibody was bought from Fuzhou Maixin Technology Development Co., Ltd., and rabbit anti-human VEGF monoclonal antibody was obtained from Beijing Zhongshan Golden Bridge Biotechnology Co., Ltd. All the secondary antibodies were purchased from DAKO, and the other reagents were obtained from Sinopharm Group Co., Ltd. (analytically pure).

### Determination of results

The expressions of COX-2, GLUT-1 and VEGF were determined as positive in the case of brownish yellow particles in the cell nucleus. Based on the proportion of positive cells, there were ≤5% as negative (-), 6%-25% as weakly positive (+), 26%-50% as moderately positive (++), and >50% as strongly positive (+++). In this study, (+)~(+++) were considered as positive expressions.

### Statistical analysis

All data were analyzed by SPSS 17.0 for windows. Relationships between the expressions of COX-2, GLUT-1 and VEGF proteins in tissues and clinical parameters were analyzed by *X*^2^ method or Fisher’s exact test. The categorical data were compared by F test, and all correlations were subjected to Spearman’s correlation analysis. P<0.05 was considered statistically significant.

## RESULTS

### Expressions of COX-2, GLUT-1 and VEGF in endometrial cancer tissues

**Fig.1:**
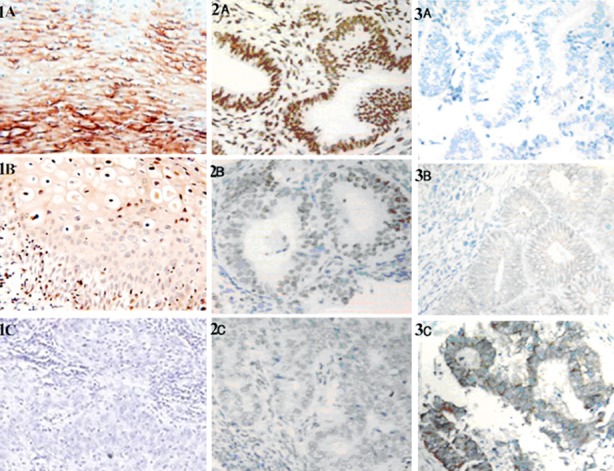
Expressions of COX-2 (1A-1C), GLUT-1 (2A-2C) and VEGF (3A-3C) proteins in cells (magnification: ×100). 1A, 2A and 3A: Normal endometrial tissues; 1B, 2B and 3B: neoplastic tissues; 1C, 2C and 3C: cancer tissues.

The expressions of the three proteins in endometrial cancer tissues are shown in Fig.1. The positive rates of COX-2 in normal group, neoplastic group and cancer groups were 3.3%, 21.7% and 55.0% respectively. The positive rates of GLUT-1 in normal group, neoplastic group and cancer groups were 3.3%, 25.0% and 70.0% respectively. The positive rates of VEGF in normal group, neoplastic group and cancer groups were 1.7%, 23.3% and 63.3% respectively. The results suggested that significantly more COX-2, GLUT-1 and VEGF were expressed with increasing degree of malignancy (P<0.05) (Table-I).

**Table-I:** Expressions of COX-2, GLUT-1 and VEGF proteins in different endometrial tissues.

Group	Protein expression			
	Case No.	COX-2 positive rate	GLUT-1 positive rate	VEGF positive rate
Normal group	60	2 (3.3%)	2 (3.3%)	1 (1.7%)
Neoplastic group	60	13 (21.7%)	15 (25.0%)	14 (23.3%)
Cancer group	60	33 (55.0%)	42 (70.0%)	38 (63.3%)
F		9.521	15.521	12.111
P		<0.05	<0.05	<0.05

### Relationships between COX-2, GLUT-1 and VEGF expressions and clinical characteristics

With increasing stage of endometrial cancer, decreasing degree of differentiation and lymphatic metastasis, the positive expression rates of COX-2, GLUT-1 and VEGF proteins increased significantly (P<0.05) (Table-II).

**Table-II:** Relationships between COX-2, GLUT-1 and VEGF expressions and clinical characteristics(n).

Variable	Case No.	COX-2 positive rate (n=33)	GLUT-1 positive rate (n=42)	VEGF positive rate (n=38)
*Staging*				
Stage I	34	16 (47.1%)	22 (64.7%)	16 (47.1%)
Stage II	20	12 (60.0%)	14 (70.0%)	16 (80.0%)
Stage III	6	5 (83.3%)	6 (100.0%)	6 (100.0%)
*Degree of differentiation*				
High	21	8 (38.1%)	12 (57.1%)	10 (47.6%)
Moderate	19	10 (52.6%)	11 (57.9%)	12 (63.2%)
Low	20	15 (75.0%)	19 (95.0%)	16 (80.0%)
*Lymphatic metastasis*				
Without	45	20 (44.4%)	28 (62.2%)	26 (57.8%)
With	15	13 (86.7%)	14 (93.3%)	12 (80.0%)

### Correlations between COX-2, GLUT-1 and VEGF expressions in endometrial cancer tissues

Spearman’s correlation analysis showed that the expressions of COX-2 and GLUT-1 (r=0.207, P<0.05), COX-2 and VEGF (r=0.243, P<0.05), as well as GLUT-1 and VEGF (r=0.758, P<0.05) were positively correlated (Table-III).

**Table-III:** Correlations between COX-2, GLUT-1 and VEGF expressions in endometrial cancer tissues.

	COX-2		GLUT-1		VEGF	
	r	P	r	P	r	P
COX-2			0.207	<0.05	0.243	<0.05
GLUT-1	0.207	<0.05			0.758	<0.05
VEGF	0.243	<0.05	0.758	<0.05		

## DISCUSSION

Endometrial cancer, as one of the most common malignant tumors in the female reproductive tract, accounts approximately for 8% of the total cases.^13^ Endometrial cancer is transformed from normal and neoplastic tissues sequentially, which is a continuous malignant transformation process that can be diagnosed based on pathological evidence. Neoplasia and cancer can be distinguished by observing the changes of endometrial glandular structures and the existence of interstitial infiltration, but not the changes of cell nuclei. The patients with early-stage endometrial cancer usually have good prognosis, but the late-stage ones suffer from poor prognosis. For instance, the 5-year survival rate of Stage-IV patients was only 10%, and metastasis also does not allow radical treatment. Therefore, it is of great significance to design an individualized treatment plan by determining stages, high-risk factors and prognostic factors.^14^

The VEGF family has six members that mainly affect the onset, proliferation and metastasis of tumors by stimulating vascularization through binding their receptors to transduce signals. In the meantime, VEGF that is secreted by tumor cells may, through the paracrine pattern, induce the neogenesis of blood vessels and lymphatic endothelial cells as well as promote the proliferation of tumor cells.^15^ Being positively expressed in normal endometrial tissues, neoplastic tissues and cancer tissues, VEGF is the most powerful promoting agent for vascular endothelial cell division and plays a critical role in the onset, invasion and metastasis of tumors.^16^ In this study, the positive expression rates of VEGF in normal, neoplastic and cancer groups were 1.7%, 23.3% and 63.3% respectively.

COX-2, which is expressed in tumor tissues as well as in new blood vessels and those with metastases, can enhance the proliferation, invasion and metastasis of tumor cells. Besides, COX-2 inhibits the anti-tumor immune response by up-regulating cell invasion.^17^ The positive expression rates of COX-2 herein were 3.3%, 21.7% and 55.0% respectively in normal, neoplastic and cancer groups, indicating that COX-2 expression increased during the pathological changes of the endometrium. Moreover, COX-2 is conducive to the migration and growth of endothelial cells as well as vascularization by stimulating tumor cells to release prostaglandin, and induces tumor angiogenesis by up-regulating related factors such as VEGF.^18^

As a member of the family of activated glucose transporter proteins, GLUT-1 predominantly controls the glucose uptake in many tumors. GLUT-1 is widely lowly expressed in normal tissues as the main carrier responsible for the transmembrane transport of glucose, but it is highly expressed in endometrial cancer, cervical squamous cell carcinoma, ovarian cancer and colon cancer.^19^ GLUT-1 is also a good marker for differentiating benign endometrium from atypical hyperplastic one, with the former having no or weak expression and the latter having different degrees of diffusive expression. Meanwhile, GLUT-1 is prone to high expressions when tumor cells are distant from the mesenchyme. Aberrant GLUT-1 expression, which may be an early event in the malignant transformation of the endometrium, provides necessary materials for its overgrowth, enables the tumor cells distant from the mesenchyme to survive, and regulates the onset of endometrial cancer. Hence, it is often employed for diagnosis and identification, which is meaningful in the prognostic evaluation on this cancer.^20^ In this study, the positive expression rates of GLUT-1 in normal, neoplastic and cancer groups were 3.3%, 25.0% and 70.0% respectively. In the meantime, the positive expression rates of COX-2, GLUT-1 and VEGF proteins increased significantly (P<0.05) with increasing stage, reducing degree of differentiation and lymphatic metastasis.

As suggested by Spearman’s correlation analysis, the expressions of COX-2 and GLUT-1 (P<0.05), COX-2 and VEGF (P<0.05), as well as GLUT-1 and VEGF (P<0.05) were positively correlated. Participating in the regulation of the MAPK signal transduction pathway, COX-2 shares homology with VEGF and activates intracellular tyrosine kinase domain after formation of dimers, thus allowing autophosphorylation of its receptors and activating reaction on the third cascade level. As a result, intranuclear transcription factors are phosphorylated and cell mitosis is promoted. The expression of GLUT-1 is positively correlated with that of VEGF, and COX-2 and VEGF both raise GLUT-1 expression via the Ras-MAPK pathway.^21^ With continuous accumulation of genetic damages, considerable anti-oncogenes are inactivated and oncogenes are activated. Given external stimulation and onset of endometrial cancer, the progression is accelerated, and tumor invasion and metastasis are further enhanced.^22^

In summary, the expressions of COX-2, GLUT-1 and VEGF were elevated in endometrial cancer to which low degree of differentiation, late stage and metastasis contributed synergistically.
